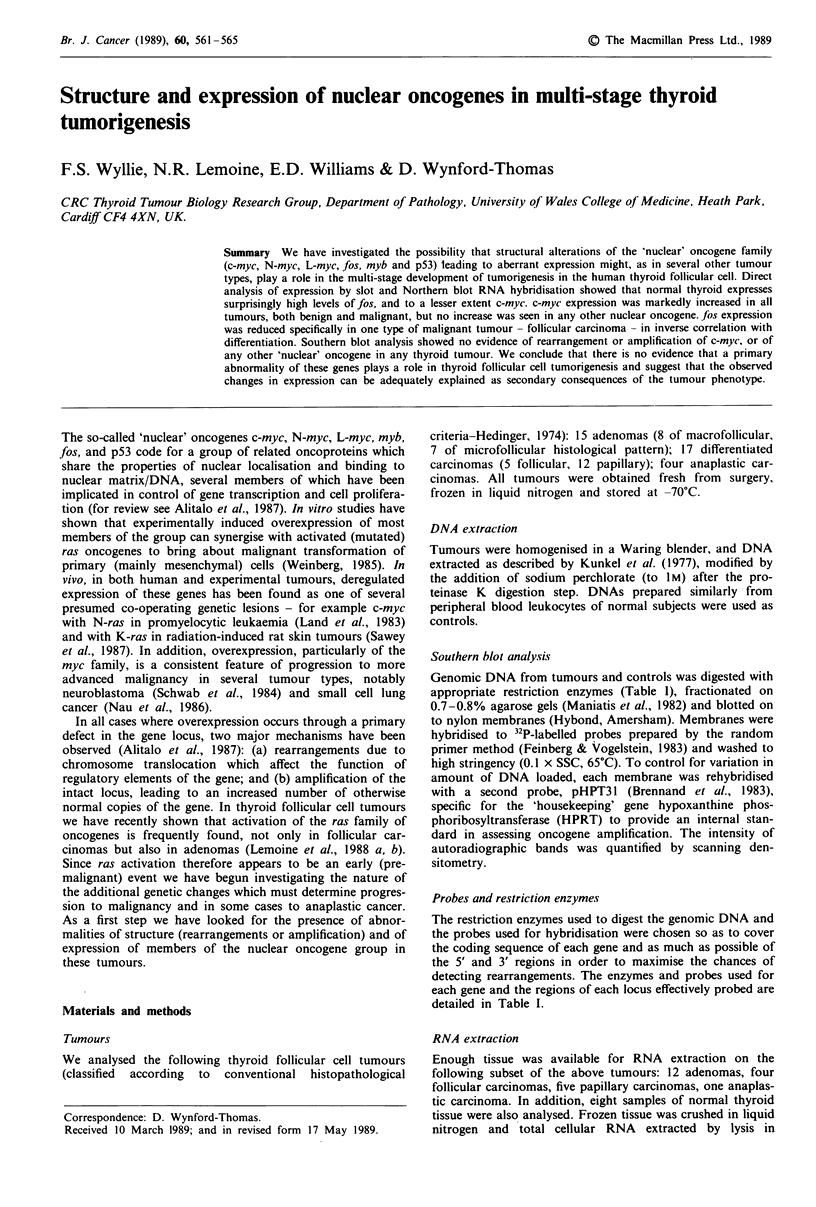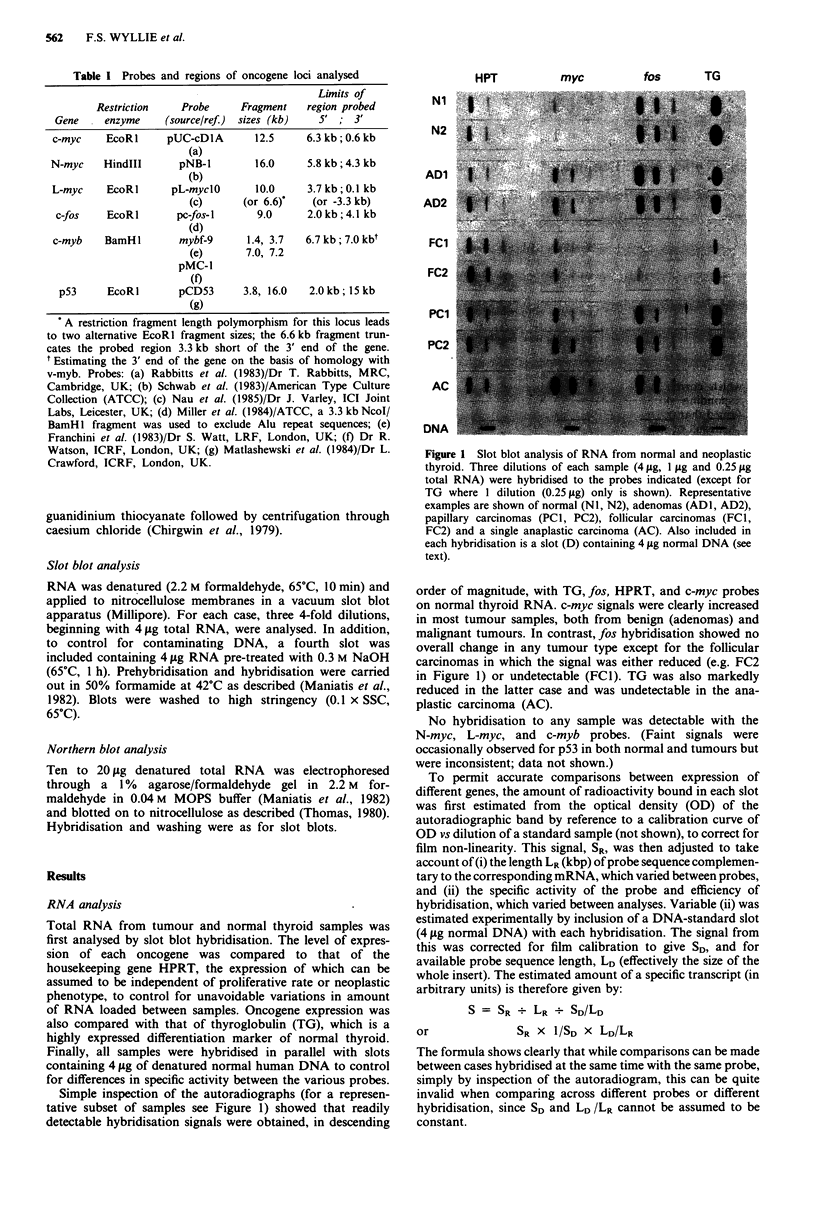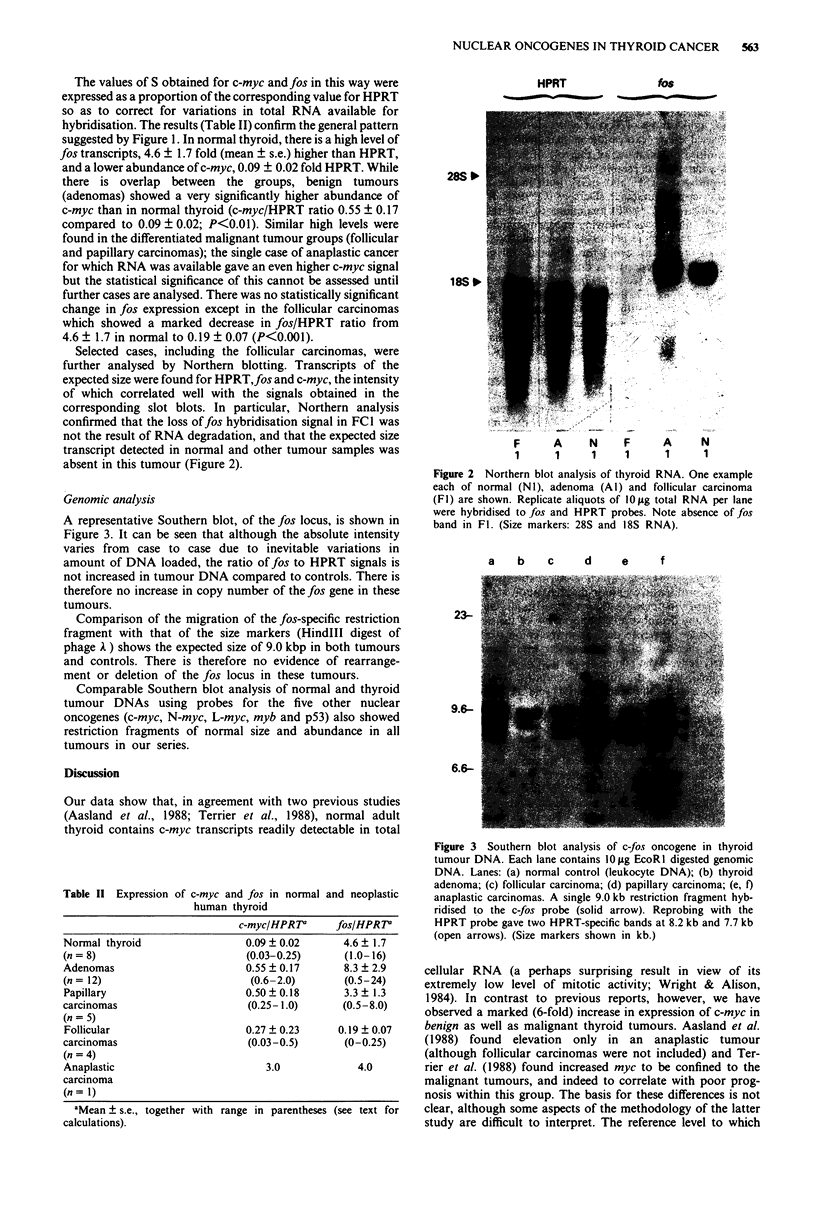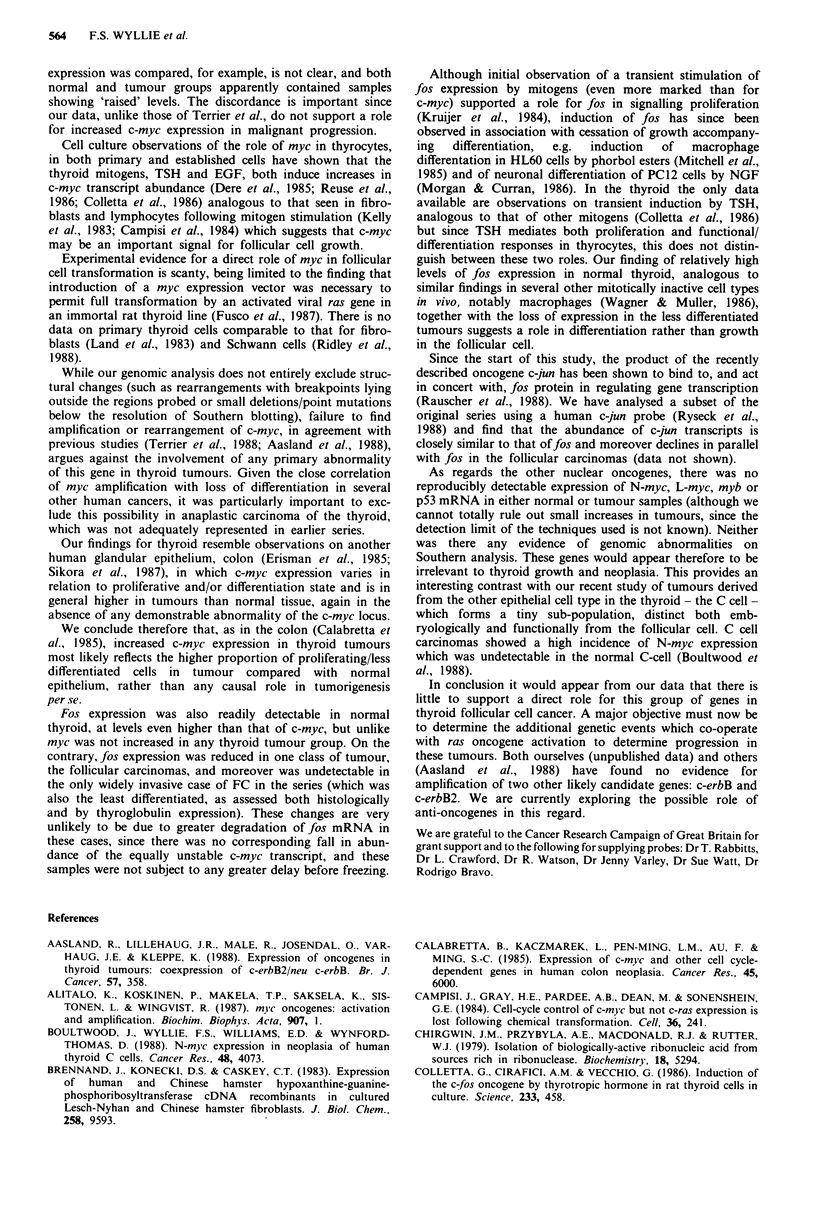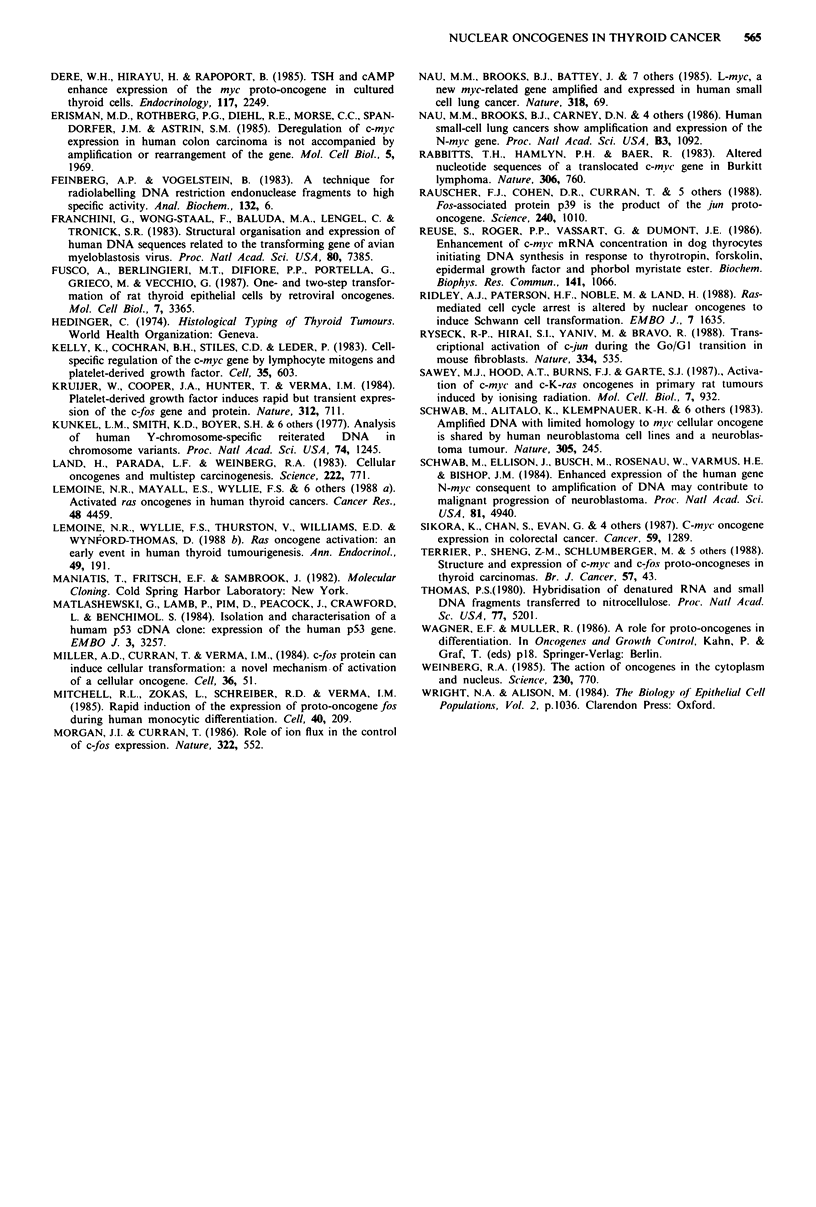# Structure and expression of nuclear oncogenes in multi-stage thyroid tumorigenesis.

**DOI:** 10.1038/bjc.1989.313

**Published:** 1989-10

**Authors:** F. S. Wyllie, N. R. Lemoine, E. D. Williams, D. Wynford-Thomas

**Affiliations:** CRC Thyroid Tumour Biology Research Group, Department of Pathology, University of Wales College of Medicine, Cardiff, UK.

## Abstract

**Images:**


Br. J. Cancer (1989), 60, 561-565 © The Macmillan Press Ltd., 1989

### Structure and expression of nuclear oncogenes in multi-stage thyroid tumorigenesis

F.S. Wyllie, N.R. Lemoine, E.D. Williams & D. Wynford-Thomas

*CRC Thyroid Tumour Biology Research Group, Department of Pathology, University of Wales College of Medicine, Heath Park, Cardiff CF4 4XN, UK.*

**Summary** We have investigated the possibility that structural alterations of the 'nuclear' oncogene family (c-*myc*, N-*myc*, L-*myc*, *fos*, *myb* and p53) leading to aberrant expression might, as in several other tumour types, play a role in the multi-stage development of tumorigenesis in the human thyroid follicular cell. Direct analysis of expression by slot and Northern blot RNA hybridisation showed that normal thyroid expresses surprisingly high levels of *fos*, and to a lesser extent c-*myc*. c-*myc* expression was markedly increased in all tumours, both benign and malignant, but no increase was seen in any other nuclear oncogene. *fos* expression was reduced specifically in one type of malignant tumour - follicular carcinoma - in inverse correlation with differentiation. Southern blot analysis showed no evidence of rearrangement or amplification of c-*myc*, or of any other 'nuclear' oncogene in any thyroid tumour. We conclude that there is no evidence that a primary abnormality of these genes plays a role in thyroid follicular cell tumorigenesis and suggest that the observed changes in expression can be adequately explained as secondary consequences of the tumour phenotype.

The so-called 'nuclear' oncogenes c-*myc*, N-*myc*, L-*myc*, *myb*, *fos*, and p53 code for a group of related oncoproteins which share the properties of nuclear localisation and binding to nuclear matrix/DNA, several members of which have been implicated in control of gene transcription and cell proliferation (for review see Alitalo *et al*., 1987). *In vitro* studies have shown that experimentally induced overexpression of most members of the group can synergise with activated (mutated) *ras* oncogenes to bring about malignant transformation of primary (mainly mesenchymal) cells (Weinberg, 1985). *In vivo*, in both human and experimental tumours, deregulated expression of these genes has been found as one of several presumed co-operating genetic lesions - for example c-*myc* with N-*ras* in promyelocytic leukaemia (Land *et al*., 1983) and with K-*ras* in radiation-induced rat skin tumours (Sawey *et al*., 1987). In addition, overexpression, particularly of the *myc* family, is a consistent feature of progression to more advanced malignancy in several tumour types, notably neuroblastoma (Schwab *et al*., 1984) and small cell lung cancer (Nau *et al*., 1986).

In all cases where overexpression occurs through a primary defect in the gene locus, two major mechanisms have been observed (Alitalo *et al*., 1987): (a) rearrangements due to chromosome translocation which affect the function of regulatory elements of the gene; and (b) amplification of the intact locus, leading to an increased number of otherwise normal copies of the gene. In thyroid follicular cell tumours we have recently shown that activation of the *ras* family of oncogenes is frequently found, not only in follicular carcinomas but also in adenomas (Lemoine *et al*., 1988 *a*, *b*). Since *ras* activation therefore appears to be an early (premalignant) event we have begun investigating the nature of the additional genetic changes which must determine progression to malignancy and in some cases to anaplastic cancer. As a first step we have looked for the presence of abnormalities of structure (rearrangements or amplification) and of expression of members of the nuclear oncogene group in these tumours.

### Materials and methods

### *Tumours*

We analysed the following thyroid follicular cell tumours (classified according to conventional histopathological criteria–Hedinger, 1974): 15 adenomas (8 of macrofollicular, 7 of microfollicular histological pattern); 17 differentiated carcinomas (5 follicular, 12 papillary); four anaplastic carcinomas. All tumours were obtained fresh from surgery, frozen in liquid nitrogen and stored at -70°C.

### *DNA extraction*

Tumours were homogenised in a Waring blender, and DNA extracted as described by Kunkel *et al*. (1977), modified by the addition of sodium perchlorate (to 1 M) after the proteinase K digestion step. DNAs prepared similarly from peripheral blood leukocytes of normal subjects were used as controls.

### *Southern blot analysis*

Genomic DNA from tumours and controls was digested with appropriate restriction enzymes (Table I), fractionated on 0.7-0.8% agarose gels (Maniatis *et al*., 1982) and blotted on to nylon membranes (Hybond, Amersham). Membranes were hybridised to $^{32}$P-labelled probes prepared by the random primer method (Feinberg & Vogelstein, 1983) and washed to high stringency (0.1 × SSC, 65°C). To control for variation in amount of DNA loaded, each membrane was rehybridised with a second probe, pHPT31 (Brennand *et al*., 1983), specific for the 'housekeeping' gene hypoxanthine phosphoribosyltransferase (HPRT) to provide an internal standard in assessing oncogene amplification. The intensity of autoradiographic bands was quantified by scanning densitometry.

### *Probes and restriction enzymes*

The restriction enzymes used to digest the genomic DNA and the probes used for hybridisation were chosen so as to cover the coding sequence of each gene and as much as possible of the 5′ and 3′ regions in order to maximise the chances of detecting rearrangements. The enzymes and probes used for each gene and the regions of each locus effectively probed are detailed in Table I.

### *RNA extraction*

Enough tissue was available for RNA extraction on the following subset of the above tumours: 12 adenomas, four follicular carcinomas, five papillary carcinomas, one anaplastic carcinoma. In addition, eight samples of normal thyroid tissue were also analysed. Frozen tissue was crushed in liquid nitrogen and total cellular RNA extracted by lysis in

Correspondence: D. Wynford-Thomas.
Received 10 March 1989; and in revised form 17 May 1989.

**Table I** Probes and regions of oncogene loci analysed

| Gene | Restriction enzyme | Probe (source/ref.) | Fragment sizes (kb) | Limits of region probed 5′ ; 3′ |
|---|---|---|---|---|
| c-*myc* | EcoR1 | pUC-cD1A (a) | 12.5 | 6.3 kb ; 0.6 kb |
| N-*myc* | HindIII | pNB-1 (b) | 16.0 | 5.8 kb ; 4.3 kb |
| L-*myc* | EcoR1 | pL-*myc*10 (c) | 10.0 (or 6.6)* | 3.7 kb ; 0.1 kb (or -3.3 kb) |
| c-*fos* | EcoR1 | pc-*fos*-1 (d) | 9.0 | 2.0 kb ; 4.1 kb |
| c-*myb* | BamH1 | *mybf*-9 (e) pMC-1 (f) | 1.4, 3.7 7.0, 7.2 | 6.7 kb ; 7.0 kb† |
| p53 | EcoR1 | pCD53 (g) | 3.8, 16.0 | 2.0 kb ; 15 kb |

*A restriction fragment length polymorphism for this locus leads to two alternative EcoR1 fragment sizes; the 6.6 kb fragment truncates the probed region 3.3 kb short of the 3′ end of the gene. †Estimating the 3′ end of the gene on the basis of homology with v-myb. Probes: (a) Rabbitts *et al.* (1983)/Dr T. Rabbitts, MRC, Cambridge, UK; (b) Schwab *et al.* (1983)/American Type Culture Collection (ATCC); (c) Nau *et al.* (1985)/Dr J. Varley, ICI Joint Labs, Leicester, UK; (d) Miller *et al.* (1984)/ATCC, a 3.3 kb NcoI/BamH1 fragment was used to exclude Alu repeat sequences; (e) Franchini *et al.* (1983)/Dr S. Watt, LRF, London, UK; (f) Dr R. Watson, ICRF, London, UK; (g) Matlashewski *et al.* (1984)/Dr L. Crawford, ICRF, London, UK.

guanidinium thiocyanate followed by centrifugation through caesium chloride (Chirgwin *et al.*, 1979).

*Slot blot analysis*

RNA was denatured (2.2 M formaldehyde, 65°C, 10 min) and applied to nitrocellulose membranes in a vacuum slot blot apparatus (Millipore). For each case, three 4-fold dilutions, beginning with 4 μg total RNA, were analysed. In addition, to control for contaminating DNA, a fourth slot was included containing 4 μg RNA pre-treated with 0.3 M NaOH (65°C, 1 h). Prehybridisation and hybridisation were carried out in 50% formamide at 42°C as described (Maniatis *et al.*, 1982). Blots were washed to high stringency (0.1 × SSC, 65°C).

*Northern blot analysis*

Ten to 20 μg denatured total RNA was electrophoresed through a 1% agarose/formaldehyde gel in 2.2 M formaldehyde in 0.04 M MOPS buffer (Maniatis *et al.*, 1982) and blotted on to nitrocellulose as described (Thomas, 1980). Hybridisation and washing were as for slot blots.

### Results

*RNA analysis*

Total RNA from tumour and normal thyroid samples was first analysed by slot blot hybridisation. The level of expression of each oncogene was compared to that of the housekeeping gene HPRT, the expression of which can be assumed to be independent of proliferative rate or neoplastic phenotype, to control for unavoidable variations in amount of RNA loaded between samples. Oncogene expression was also compared with that of thyroglobulin (TG), which is a highly expressed differentiation marker of normal thyroid. Finally, all samples were hybridised in parallel with slots containing 4 μg of denatured normal human DNA to control for differences in specific activity between the various probes.

Simple inspection of the autoradiographs (for a representative subset of samples see Figure 1) showed that readily detectable hybridisation signals were obtained, in descending order of magnitude, with TG, *fos*, HPRT, and c-*myc* probes on normal thyroid RNA. c-*myc* signals were clearly increased in most tumour samples, both from benign (adenomas) and malignant tumours. In contrast, *fos* hybridisation showed no overall change in any tumour type except for the follicular carcinomas in which the signal was either reduced (e.g. FC2 in Figure 1) or undetectable (FC1). TG was also markedly reduced in the latter case and was undetectable in the anaplastic carcinoma (AC).

**Figure 1** Slot blot analysis of RNA from normal and neoplastic thyroid. Three dilutions of each sample (4 μg, 1 μg and 0.25 μg total RNA) were hybridised to the probes indicated (except for TG where 1 dilution (0.25 μg) only is shown). Representative examples are shown of normal (N1, N2), adenomas (AD1, AD2), papillary carcinomas (PC1, PC2), follicular carcinomas (FC1, FC2) and a single anaplastic carcinoma (AC). Also included in each hybridisation is a slot (D) containing 4 μg normal DNA (see text).

No hybridisation to any sample was detectable with the N-*myc*, L-*myc*, and c-*myb* probes. (Faint signals were occasionally observed for p53 in both normal and tumours but were inconsistent; data not shown.)

To permit accurate comparisons between expression of different genes, the amount of radioactivity bound in each slot was first estimated from the optical density (OD) of the autoradiographic band by reference to a calibration curve of OD *vs* dilution of a standard sample (not shown), to correct for film non-linearity. This signal, $S_R$, was then adjusted to take account of (i) the length $L_R$ (kbp) of probe sequence complementary to the corresponding mRNA, which varied between probes, and (ii) the specific activity of the probe and efficiency of hybridisation, which varied between analyses. Variable (ii) was estimated experimentally by inclusion of a DNA-standard slot (4 μg normal DNA) with each hybridisation. The signal from this was corrected for film calibration to give $S_D$, and for available probe sequence length, $L_D$ (effectively the size of the whole insert). The estimated amount of a specific transcript (in arbitrary units) is therefore given by:

$$S = S_R \div L_R \div S_D/L_D$$

or

$$S_R \times 1/S_D \times L_D/L_R$$

The formula shows clearly that while comparisons can be made between cases hybridised at the same time with the same probe, simply by inspection of the autoradiogram, this can be quite invalid when comparing across different probes or different hybridisation, since $S_D$ and $L_D/L_R$ cannot be assumed to be constant.

The values of S obtained for c-*myc* and *fos* in this way were expressed as a proportion of the corresponding value for HPRT so as to correct for variations in total RNA available for hybridisation. The results (Table II) confirm the general pattern suggested by Figure 1. In normal thyroid, there is a high level of *fos* transcripts, 4.6 ± 1.7 fold (mean ± s.e.) higher than HPRT, and a lower abundance of c-*myc*, 0.09 ± 0.02 fold HPRT. While there is overlap between the groups, benign tumours (adenomas) showed a very significantly higher abundance of c-*myc* than in normal thyroid (c-*myc*/HPRT ratio 0.55 ± 0.17 compared to 0.09 ± 0.02; $P<0.01$). Similar high levels were found in the differentiated malignant tumour groups (follicular and papillary carcinomas); the single case of anaplastic cancer for which RNA was available gave an even higher c-*myc* signal but the statistical significance of this cannot be assessed until further cases are analysed. There was no statistically significant change in *fos* expression except in the follicular carcinomas which showed a marked decrease in *fos*/HPRT ratio from 4.6 ± 1.7 in normal to 0.19 ± 0.07 ($P<0.001$).

Selected cases, including the follicular carcinomas, were further analysed by Northern blotting. Transcripts of the expected size were found for HPRT, *fos* and c-*myc*, the intensity of which correlated well with the signals obtained in the corresponding slot blots. In particular, Northern analysis confirmed that the loss of *fos* hybridisation signal in FC1 was not the result of RNA degradation, and that the expected size transcript detected in normal and other tumour samples was absent in this tumour (Figure 2).

*Genomic analysis*

A representative Southern blot, of the *fos* locus, is shown in Figure 3. It can be seen that although the absolute intensity varies from case to case due to inevitable variations in amount of DNA loaded, the ratio of *fos* to HPRT signals is not increased in tumour DNA compared to controls. There is therefore no increase in copy number of the *fos* gene in these tumours.

Comparison of the migration of the *fos*-specific restriction fragment with that of the size markers (HindIII digest of phage λ) shows the expected size of 9.0 kbp in both tumours and controls. There is therefore no evidence of rearrangement or deletion of the *fos* locus in these tumours.

Comparable Southern blot analysis of normal and thyroid tumour DNAs using probes for the five other nuclear oncogenes (c-*myc*, N-*myc*, L-*myc*, *myb* and p53) also showed restriction fragments of normal size and abundance in all tumours in our series.

### Discussion

Our data show that, in agreement with two previous studies (Aasland *et al.*, 1988; Terrier *et al.*, 1988), normal adult thyroid contains c-*myc* transcripts readily detectable in total cellular RNA (a perhaps surprising result in view of its extremely low level of mitotic activity; Wright & Alison, 1984). In contrast to previous reports, however, we have observed a marked (6-fold) increase in expression of c-*myc* in *benign* as well as malignant thyroid tumours. Aasland *et al.* (1988) found elevation only in an anaplastic tumour (although follicular carcinomas were not included) and Terrier *et al.* (1988) found increased *myc* to be confined to the malignant tumours, and indeed to correlate with poor prognosis within this group. The basis for these differences is not clear, although some aspects of the methodology of the latter study are difficult to interpret. The reference level to which

**Table II** Expression of c-*myc* and *fos* in normal and neoplastic human thyroid

| | c-*myc*/*HPRT*[a] | *fos*/*HPRT*[a] |
|---|---|---|
| Normal thyroid ($n$ = 8) | 0.09 ± 0.02 (0.03–0.25) | 4.6 ± 1.7 (1.0–16) |
| Adenomas ($n$ = 12) | 0.55 ± 0.17 (0.6–2.0) | 8.3 ± 2.9 (0.5–24) |
| Papillary carcinomas ($n$ = 5) | 0.50 ± 0.18 (0.25–1.0) | 3.3 ± 1.3 (0.5–8.0) |
| Follicular carcinomas ($n$ = 4) | 0.27 ± 0.23 (0.03–0.5) | 0.19 ± 0.07 (0–0.25) |
| Anaplastic carcinoma ($n$ = 1) | 3.0 | 4.0 |

[a]Mean ± s.e., together with range in parentheses (see text for calculations).

**Figure 2** Northern blot analysis of thyroid RNA. One example each of normal (N1), adenoma (A1) and follicular carcinoma (F1) are shown. Replicate aliquots of 10 µg total RNA per lane were hybridised to *fos* and HPRT probes. Note absence of *fos* band in F1. (Size markers: 28S and 18S RNA).

**Figure 3** Southern blot analysis of c-*fos* oncogene in thyroid tumour DNA. Each lane contains 10 µg EcoR1 digested genomic DNA. Lanes: (a) normal control (leukocyte DNA); (b) thyroid adenoma; (c) follicular carcinoma; (d) papillary carcinoma; (e, f) anaplastic carcinomas. A single 9.0 kb restriction fragment hybridised to the c-*fos* probe (solid arrow). Reprobing with the HPRT probe gave two HPRT-specific bands at 8.2 kb and 7.7 kb (open arrows). (Size markers shown in kb.)

expression was compared, for example, is not clear, and both normal and tumour groups apparently contained samples showing 'raised' levels. The discordance is important since our data, unlike those of Terrier *et al.*, do not support a role for increased c-*myc* expression in malignant progression.

Cell culture observations of the role of *myc* in thyrocytes, in both primary and established cells have shown that the thyroid mitogens, TSH and EGF, both induce increases in c-*myc* transcript abundance (Dere *et al.*, 1985; Reuse *et al.*, 1986; Colletta *et al.*, 1986) analogous to that seen in fibroblasts and lymphocytes following mitogen stimulation (Kelly *et al.*, 1983; Campisi *et al.*, 1984) which suggests that c-*myc* may be an important signal for follicular cell growth.

Experimental evidence for a direct role of *myc* in follicular cell transformation is scanty, being limited to the finding that introduction of a *myc* expression vector was necessary to permit full transformation by an activated viral *ras* gene in an immortal rat thyroid line (Fusco *et al.*, 1987). There is no data on primary thyroid cells comparable to that for fibroblasts (Land *et al.*, 1983) and Schwann cells (Ridley *et al.*, 1988).

While our genomic analysis does not entirely exclude structural changes (such as rearrangements with breakpoints lying outside the regions probed or small deletions/point mutations below the resolution of Southern blotting), failure to find amplification or rearrangement of c-*myc*, in agreement with previous studies (Terrier *et al.*, 1988; Aasland *et al.*, 1988), argues against the involvement of any primary abnormality of this gene in thyroid tumours. Given the close correlation of *myc* amplification with loss of differentiation in several other human cancers, it was particularly important to exclude this possibility in anaplastic carcinoma of the thyroid, which was not adequately represented in earlier series.

Our findings for thyroid resemble observations on another human glandular epithelium, colon (Erisman *et al.*, 1985; Sikora *et al.*, 1987), in which c-*myc* expression varies in relation to proliferative and/or differentiation state and is in general higher in tumours than normal tissue, again in the absence of any demonstrable abnormality of the c-*myc* locus.

We conclude therefore that, as in the colon (Calabretta *et al.*, 1985), increased c-*myc* expression in thyroid tumours most likely reflects the higher proportion of proliferating/less differentiated cells in tumour compared with normal epithelium, rather than any causal role in tumorigenesis *per se*.

*Fos* expression was also readily detectable in normal thyroid, at levels even higher than that of c-*myc*, but unlike *myc* was not increased in any thyroid tumour group. On the contrary, *fos* expression was reduced in one class of tumour, the follicular carcinomas, and moreover was undetectable in the only widely invasive case of FC in the series (which was also the least differentiated, as assessed both histologically and by thyroglobulin expression). These changes are very unlikely to be due to greater degradation of *fos* mRNA in these cases, since there was no corresponding fall in abundance of the equally unstable c-*myc* transcript, and these samples were not subject to any greater delay before freezing.

Although initial observation of a transient stimulation of *fos* expression by mitogens (even more marked than for c-*myc*) supported a role for *fos* in signalling proliferation (Kruijer *et al.*, 1984), induction of *fos* has since been observed in association with cessation of growth accompanying differentiation, e.g. induction of macrophage differentation in HL60 cells by phorbol esters (Mitchell *et al.*, 1985) and of neuronal differentiation of PC12 cells by NGF (Morgan & Curran, 1986). In the thyroid the only data available are observations on transient induction by TSH, analogous to that of other mitogens (Colletta *et al.*, 1986) but since TSH mediates both proliferation and functional/differentiation responses in thyrocytes, this does not distinguish between these two roles. Our finding of relatively high levels of *fos* expression in normal thyroid, analogous to similar findings in several other mitotically inactive cell types *in vivo*, notably macrophages (Wagner & Muller, 1986), together with the loss of expression in the less differentiated tumours suggests a role in differentiation rather than growth in the follicular cell.

Since the start of this study, the product of the recently described oncogene c-*jun* has been shown to bind to, and act in concert with, *fos* protein in regulating gene transcription (Rauscher *et al.*, 1988). We have analysed a subset of the original series using a human c-*jun* probe (Ryseck *et al.*, 1988) and find that the abundance of c-*jun* transcripts is closely similar to that of *fos* and moreover declines in parallel with *fos* in the follicular carcinomas (data not shown).

As regards the other nuclear oncogenes, there was no reproducibly detectable expression of N-*myc*, L-*myc*, *myb* or p53 mRNA in either normal or tumour samples (although we cannot totally rule out small increases in tumours, since the detection limit of the techniques used is not known). Neither was there any evidence of genomic abnormalities on Southern analysis. These genes would appear therefore to be irrelevant to thyroid growth and neoplasia. This provides an interesting contrast with our recent study of tumours derived from the other epithelial cell type in the thyroid – the C cell – which forms a tiny sub-population, distinct both embryologically and functionally from the follicular cell. C cell carcinomas showed a high incidence of N-*myc* expression which was undetectable in the normal C-cell (Boultwood *et al.*, 1988).

In conclusion it would appear from our data that there is little to support a direct role for this group of genes in thyroid follicular cell cancer. A major objective must now be to determine the additional genetic events which co-operate with *ras* oncogene activation to determine progression in these tumours. Both ourselves (unpublished data) and others (Aasland *et al.*, 1988) have found no evidence for amplification of two other likely candidate genes: c-*erb*B and c-*erb*B2. We are currently exploring the possible role of anti-oncogenes in this regard.

We are grateful to the Cancer Research Campaign of Great Britain for grant support and to the following for supplying probes: Dr T. Rabbitts, Dr L. Crawford, Dr R. Watson, Dr Jenny Varley, Dr Sue Watt, Dr Rodrigo Bravo.

### References

AASLAND, R., LILLEHAUG, J.R., MALE, R., JOSENDAL, O., VARHAUG, J.E. & KLEPPE, K. (1988). Expression of oncogenes in thyroid tumours: coexpression of c-*erb*B2/*neu* c-*erb*B. *Br. J. Cancer*, **57**, 358.

ALITALO, K., KOSKINEN, P., MAKELA, T.P., SAKSELA, K., SISTONEN, L. & WINGVIST, R. (1987). *myc* oncogenes: activation and amplification. *Biochim. Biophys. Acta*, **907**, 1.

BOULTWOOD, J., WYLLIE, F.S., WILLIAMS, E.D. & WYNFORD-THOMAS, D. (1988). N-*myc* expression in neoplasia of human thyroid C cells. *Cancer Res.*, **48**, 4073.

BRENNAND, J., KONECKI, D.S. & CASKEY, C.T. (1983). Expression of human and Chinese hamster hypoxanthine-guanine-phosphoribosyltransferase cDNA recombinants in cultured Lesch-Nyhan and Chinese hamster fibroblasts. *J. Biol. Chem.*, **258**, 9593.

CALABRETTA, B., KACZMAREK, L., PEN-MING, L.M., AU, F. & MING, S.-C. (1985). Expression of c-*myc* and other cell cycle-dependent genes in human colon neoplasia. *Cancer Res.*, **45**, 6000.

CAMPISI, J., GRAY, H.E., PARDEE, A.B., DEAN, M. & SONENSHEIN, G.E. (1984). Cell-cycle control of c-*myc* but not c-*ras* expression is lost following chemical transformation. *Cell*, **36**, 241.

CHIRGWIN, J.M., PRZYBYLA, A.E., MACDONALD, R.J. & RUTTER, W.J. (1979). Isolation of biologically-active ribonucleic acid from sources rich in ribonuclease. *Biochemistry*, **18**, 5294.

COLLETTA, G., CIRAFICI, A.M. & VECCHIO, G. (1986). Induction of the c-*fos* oncogene by thyrotropic hormone in rat thyroid cells in culture. *Science*, **233**, 458.

DERE, W.H., HIRAYU, H. & RAPOPORT, B. (1985). TSH and cAMP enhance expression of the *myc* proto-oncogene in cultured thyroid cells. *Endocrinology*, **117**, 2249.

ERISMAN, M.D., ROTHBERG, P.G., DIEHL, R.E., MORSE, C.C., SPANDORFER, J.M. & ASTRIN, S.M. (1985). Deregulation of c-*myc* expression in human colon carcinoma is not accompanied by amplification or rearrangement of the gene. *Mol. Cell Biol.*, **5**, 1969.

FEINBERG, A.P. & VOGELSTEIN, B. (1983). A technique for radiolabelling DNA restriction endonuclease fragments to high specific activity. *Anal. Biochem.*, **132**, 6.

FRANCHINI, G., WONG-STAAL, F., BALUDA, M.A., LENGEL, C. & TRONICK, S.R. (1983). Structural organisation and expression of human DNA sequences related to the transforming gene of avian myeloblastosis virus. *Proc. Natl Acad. Sci. USA*, **80**, 7385.

FUSCO, A., BERLINGIERI, M.T., DIFIORE, P.P., PORTELLA, G., GRIECO, M. & VECCHIO, G. (1987). One- and two-step transformation of rat thyroid epithelial cells by retroviral oncogenes. *Mol. Cell Biol.*, **7**, 3365.

HEDINGER, C. (1974). *Histological Typing of Thyroid Tumours*. World Health Organization: Geneva.

KELLY, K., COCHRAN, B.H., STILES, C.D. & LEDER, P. (1983). Cell-specific regulation of the c-*myc* gene by lymphocyte mitogens and platelet-derived growth factor. *Cell*, **35**, 603.

KRUIJER, W., COOPER, J.A., HUNTER, T. & VERMA, I.M. (1984). Platelet-derived growth factor induces rapid but transient expression of the c-*fos* gene and protein. *Nature*, **312**, 711.

KUNKEL, L.M., SMITH, K.D., BOYER, S.H. & 6 others (1977). Analysis of human Y-chromosome-specific reiterated DNA in chromosome variants. *Proc. Natl Acad. Sci. USA*, **74**, 1245.

LAND, H., PARADA, L.F. & WEINBERG, R.A. (1983). Cellular oncogenes and multistep carcinogenesis. *Science*, **222**, 771.

LEMOINE, N.R., MAYALL, E.S., WYLLIE, F.S. & 6 others (1988 *a*). Activated *ras* oncogenes in human thyroid cancers. *Cancer Res.*, **48** 4459.

LEMOINE, N.R., WYLLIE, F.S., THURSTON, V., WILLIAMS, E.D. & WYNFORD-THOMAS, D. (1988 *b*). *Ras* oncogene activation: an early event in human thyroid tumourigenesis. *Ann. Endocrinol.*, **49**, 191.

MANIATIS, T., FRITSCH, E.F. & SAMBROOK, J. (1982). *Molecular Cloning*. Cold Spring Harbor Laboratory: New York.

MATLASHEWSKI, G., LAMB, P., PIM, D., PEACOCK, J., CRAWFORD, L. & BENCHIMOL. S. (1984). Isolation and characterisation of a humam p53 cDNA clone: expression of the human p53 gene. *EMBO J.* **3**, 3257.

MILLER, A.D., CURRAN, T. & VERMA, I.M., (1984). c-*fos* protein can induce cellular transformation: a novel mechanism of activation of a cellular oncogene. *Cell*, **36**, 51.

MITCHELL, R.L., ZOKAS, L., SCHREIBER, R.D. & VERMA, I.M. (1985). Rapid induction of the expression of proto-oncogene *fos* during human monocytic differentiation. *Cell*, **40**, 209.

MORGAN, J.I. & CURRAN, T. (1986). Role of ion flux in the control of c-*fos* expression. *Nature*, **322**, 552.

NAU, M.M., BROOKS, B.J., BATTEY, J. & 7 others (1985). L-*myc*, a new *myc*-related gene amplified and expressed in human small cell lung cancer. *Nature*, **318**, 69.

NAU, M.M., BROOKS, B.J., CARNEY, D.N. & 4 others (1986). Human small-cell lung cancers show amplification and expression of the N-*myc* gene. *Proc. Natl Acad. Sci. USA*, **B3**, 1092.

RABBITTS, T.H., HAMLYN, P.H. & BAER, R. (1983). Altered nucleotide sequences of a translocated c-*myc* gene in Burkitt lymphoma. *Nature*, **306**, 760.

RAUSCHER, F.J., COHEN, D.R., CURRAN, T. & 5 others (1988). *Fos*-associated protein p39 is the product of the *jun* proto-oncogene. *Science*, **240**, 1010.

REUSE, S., ROGER, P.P., VASSART, G. & DUMONT, J.E. (1986). Enhancement of c-*myc* mRNA concentration in dog thyrocytes initiating DNA synthesis in response to thyrotropin, forskolin, epidermal growth factor and phorbol myristate ester. *Biochem. Biophys. Res. Commun.*, **141**, 1066.

RIDLEY, A.J., PATERSON, H.F., NOBLE, M. & LAND, H. (1988). *Ras*-mediated cell cycle arrest is altered by nuclear oncogenes to induce Schwann cell transformation. *EMBO J.*, **7** 1635.

RYSECK, R-P., HIRAI, S.I., YANIV, M. & BRAVO, R. (1988). Transcriptional activation of c-*jun* during the Go/G1 transition in mouse fibroblasts. *Nature*, **334**, 535.

SAWEY, M.J., HOOD, A.T., BURNS, F.J. & GARTE, S.J. (1987)., Activation of c-*myc* and c-K-*ras* oncogenes in primary rat tumours induced by ionising radiation. *Mol. Cell. Biol.*, **7**, 932.

SCHWAB, M., ALITALO, K., KLEMPNAUER, K-H. & 6 others (1983). Amplified DNA with limited homology to *myc* cellular oncogene is shared by human neuroblastoma cell lines and a neuroblastoma tumour. *Nature*, **305**, 245.

SCHWAB, M., ELLISON, J., BUSCH, M., ROSENAU, W., VARMUS, H.E. & BISHOP, J.M. (1984). Enhanced expression of the human gene N-*myc* consequent to amplification of DNA may contribute to malignant progression of neuroblastoma. *Proc. Natl Acad. Sci. USA*, **81**, 4940.

SIKORA, K., CHAN, S., EVAN, G. & 4 others (1987). C-*myc* oncogene expression in colorectal cancer. *Cancer*, **59**, 1289.

TERRIER, P., SHENG, Z-M., SCHLUMBERGER, M. & 5 others (1988). Structure and expression of c-*myc* and c-*fos* proto-oncogneses in thyroid carcinomas. *Br. J. Cancer*, **57**, 43.

THOMAS, P.S.(1980). Hybridisation of denatured RNA and small DNA fragments transferred to nitrocellulose. *Proc. Natl Acad. Sc. USA*, **77**, 5201.

WAGNER, E.F. & MULLER, R. (1986). A role for proto-oncogenes in differentiation. In *Oncogenes and Growth Control*, Kahn, P. & Graf, T. (eds) p18. Springer-Verlag: Berlin.

WEINBERG, R.A. (1985). The action of oncogenes in the cytoplasm and nucleus. *Science*, **230**, 770.

WRIGHT, N.A. & ALISON, M. (1984). *The Biology of Epithelial Cell Populations, Vol. 2*, p.1036. Clarendon Press: Oxford.